# Fluorescent detection of peritoneal metastasis in human colorectal cancer using 5-aminolevulinic acid

**DOI:** 10.3892/ijo.2014.2417

**Published:** 2014-05-06

**Authors:** YUTAKA KONDO, YASUTOSHI MURAYAMA, HIROTAKA KONISHI, RYO MORIMURA, SHUHEI KOMATSU, ATSUSHI SHIOZAKI, YOSHIAKI KURIU, HISASHI IKOMA, TAKESHI KUBOTA, MASAYOSHI NAKANISHI, DAISUKE ICHIKAWA, HITOSHI FUJIWARA, KAZUMA OKAMOTO, CHOUHEI SAKAKURA, KIWAMU TAKAHASHI, KATSUSHI INOUE, MOTOWO NAKAJIMA, EIGO OTSUJI

**Affiliations:** 1 Division of Digestive Surgery, Department of Surgery, Kyoto Prefectural University of Medicine, Kyoto 602-8566; 2 SBI Pharmaceuticals Co., Ltd., Minato-ku, Tokyo 106-6019, Japan

**Keywords:** 5-aminolevulinic acid, photodynamic diagnosis, colorectal cancer, peritoneal metastasis, protoporphyrin IX, laparoscopy

## Abstract

A precise diagnosis of peritoneal dissemination is necessary to determine the appropriate treatment strategy for colorectal cancer. However, small peritoneal dissemination is difficult to diagnose. 5-aminolevulinic acid (5-ALA) is an intermediate substrate of heme metabolism. The administration of 5-ALA to cancer patients results in tumor-specific accumulation of protoporphyrin IX (PpIX), which emits red fluorescence with blue light irradiation. We evaluated the usefulness of photodynamic diagnosis (PDD) using 5-ALA to detect the peritoneal dissemination of colorectal cancer. EGFP-tagged HT-29 cells were injected into the peritoneal cavity of BALB/c nude mice. After 2 weeks, the mice were given 5-ALA hydrochloride, and metastatic nodules in the omentum were observed with white light and fluorescence images. Twelve colorectal cancer patients suspected to have serosal invasion according to preoperative computed tomography (CT) were enrolled in this study. 5-ALA (15-20 mg per kg body weight) was administered orally to the patients 3 h before surgery. The abdominal cavity was observed under white light and fluorescence. Fluorescence images were analyzed with image analysis software (ImageJ 1.45s, National Institutes of Health, Bethesda, MD, USA). The mice developed peritoneal disseminations. The observed 5-ALA-induced red fluorescence was consistent with the EGFP fluorescent-positive nodules. Peritoneal dissemination was observed with conventional white light imaging in 8 patients. All nodules suspected as being peritoneal dissemination lesions by white light observation were similarly detected by ALA-induced fluorescence. In 1 patient, a small, flat lesion that was missed under white light observation was detected by ALA-induced fluorescence; the lesion was pathologically diagnosed as peritoneal metastasis. In the quantitative fluorescence image analysis, the red/(red + green + blue) ratio was higher in the metastatic nodules compared to the non-metastatic sites of the abdominal wall, fat and liver. We demonstrated better diagnostic accuracy using 5-ALA-PDD compared to conventional laparoscopy in patients with colorectal cancer. 5-ALA-PDD is a promising candidate method for diagnosing peritoneal dissemination of colorectal cancer.

## Introduction

Colorectal cancer is the third most common cancer worldwide. More than 1 million people develop colorectal cancer. Distant metastasis is one of the most important prognostic factors for determining how patients will respond to colorectal cancer treatment. Recently, the survival rates for colorectal cancer patients have improved dramatically because of earlier diagnoses and advances in anticancer therapy, such as molecular-targeted agents. Prognostic improvement is anticipated even for colorectal cancer patients who have peritoneal dissemination, particularly if all of the nodes are surgically removed or if the patient is given chemotherapy at an appropriate time (1–4). However, diagnosing the peritoneal dissemination is difficult. The peritoneal dissemination of small nodules is not detectable on pre-operative computed tomography (CT) or ^18^F-fluoro-deoxy-glucose positron emission tomography (FDG-PET). Bamba *et al* have reported that the sensitivity of FDG-PET/CT for colorectal cancer peritoneal dissemination is 82.6% (5). Therefore, small lesions can only be diagnosed by intra-operative findings, or they are sometimes missed during surgery. Therefore, the precise diagnosis of peritoneal dissemination is necessary to determine the appropriate treatment strategy for colorectal cancer.

In this study, we evaluated the usefulness of photodynamic diagnosis (PDD) using 5-aminolevulinic acid (5-ALA) to detect peritoneal dissemination of colorectal cancer. 5-ALA is a natural precursor of the heme. In cancer cells, increased activity of porphobilinogen deaminase and decreased activity of ferrochelatase cause the intracellular accumulation of protoporphyrin IX (PpIX) (6). PpIX emits red fluorescence and peaks at 635 nm, with a blue-violet light excitation of 405 nm. Based on these mechanisms, 5-ALA has been used clinically as a photosensitizer in PDD in neurosurgery and urology (7–10). Previous reports on 5-ALA have demonstrated improved diagnostic performances in these fields. Moreover, 5-ALA is also used in photodynamic therapy (PDT) (11,12).

Recently, we reported on the efficacy of 5-ALA for detecting lymph node metastasis of rectal cancer in mouse models (13). Additionally, we previously reported on the diagnostic usefulness of using 5-ALA for peritoneal dissemination and lymph node metastasis in gastric cancer patients (14,15). In this study, we applied this method to fluorescent laparoscopy for detecting peritoneal dissemination of human colorectal cancers, and we compared the diagnostic accuracy of 5-ALA use with conventional laparoscopy in the clinical setting.

## Materials and methods

### Cell line and cell culture

The human colorectal cancer cell line HT-29 was used. HT-29 was cultured in McCoy’s medium with 10% fetal bovine serum, 100 U/ml penicillin, and 100 *μ*g/ml streptomycin at 37°C in a water-saturated atmosphere with 5% CO_2_/95% air. The HT-29 cell line stably expressing enhanced green fluorescent protein (EGFP) was established as previously described (13). Briefly, HT-29 cells were transiently transfected with an EGFP plasmid, and the successfully transfected cells were then selected using 1 mg/ml G418 (Wako Pure Chemical Industries, Osaka, Japan).

### Animals

Five-week-old female BALB/c nude mice were used in this study. All mice were housed in groups in plastic cages with stainless-steel grid tops in an air-conditioned environment with a 12-h light-dark cycle. The mice were given *ad libitum* access to food and water. All animal experiments were approved and followed the institutional guidelines of the Kyoto Prefectural University of Medicine.

### Establishment of the mouse model of peritoneal metastasis and fluorescent observation

An aliquot of 1×10^6^ EGFP tagged HT-29 cells was injected into the peritoneal cavity of mice under general anesthesia. After 2 weeks, the mice were intraperitoneally injected with 5-ALA hydrochloride (Wako Pure Chemical Industries, Osaka, Japan) at a dose of 250 mg/kg body weight. Six hours after 5-ALA administration, the mice were euthanized and laparotomy was performed. Metastatic nodules in the omentum were observed in white light and fluorescence images. Fluorescence observation was performed with a stereoscopic microscope (SZX12; Olympus, Tokyo, Japan) equipped with a color CCD digital camera (DP71, Olympus) and a mercury lamp (U-LH100HG; Olympus). We used a spectral analytic system composed of a stereoscopic microscope equipped with an intensified multi-channel spectrophotometer (MCPD-7000, Otsuka Electronics, Osaka, Japan) for spectral analysis. PpIX images (>430 nm, HQ430LP, Chroma Technology Corp., Rockingham, VT, USA) were acquired by exciting at 405±20 nm (D405/20x, Chroma Technology Corp.), and EGFP fluorescence images (510–530 nm) (GFPA cube, Olympus) were acquired by exciting at 460–490 nm (GFPA cube, Olympus); all images were recorded in the red, green and blue format.

### Enrolled patients

A clinical trial was conducted from March 2011 to March 2013 with approval from the Ethics Committee of Kyoto Prefectural University of Medicine, Kyoto, Japan. Twelve colorectal cancer patients suspected of having serosal invasion (by preoperative CT scanning) were included in the study. The patients provided signed informed consent preoperatively. The exclusion criteria included the presence of porphyria and obstruction of the digestive tract. The clinical findings were categorized according to the UICC 7th TNM classification.

### Laparoscopic procedure

5-ALA hydrochloride (Cosmo Bio Co., Ltd., Tokyo, Japan) dissolved in 20 ml of 50% glucose solution was orally administered 3 h before surgery at a dose of 15–20 mg per kg body weight, ≤1 g per patient. The system used for the fluorescence laparoscopic analyses consisted of a laparoscopic videoscope (OTV-Y0007, Olympus) equipped with a video system center (EVIS EXCERAII CV-180, Olympus) and a xenon light source (EVIS EXCERAII CLV-180, Olympus). At the start of the laparoscopic surgery, the abdominal cavity was observed in white light and fluorescence images with a long pass filter (>450 nm); images were taken under excitation with blue-violet light (380–430 nm). Grossly apparent peritoneal dissemination nodules were omitted from the sampling.

The final four patients (nos. 9–12) were observed with a D-LIGHT system (Karl Storz GmbH & Co., Tuttlingen, Germany). They were excluded from the quantitative analysis.

### Quantitative analysis

Fluorescence images were analyzed with image analysis software (ImageJ 1.45s, National Institutes of Health, Bethesda, MD, USA). The red value of the 24-bit RGB color image was evaluated as a corresponding index for red fluorescence. The maximum red value of each peritoneal metastatic nodule and non-metastatic site of the abdominal wall, fat, and liver were compared. The red/(red + green + blue) ratio was also evaluated to correct for differences in the imaging conditions.

## Results

### Mouse model

**Figure 1.**
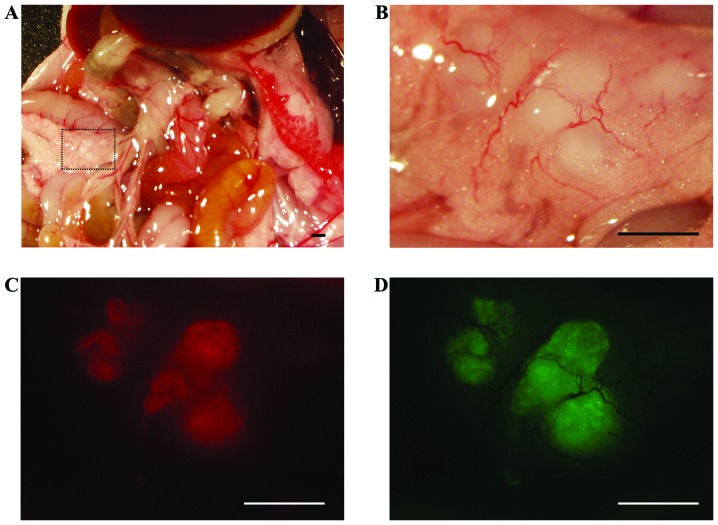
Imaging of peritoneal metastases with 5-ALA in a xenograft mouse model with human colorectal cancer cells. (A) A microscopic image of the abdominal cavity of the mouse 5 weeks after the HT-29/EGFP cell implantation. (B) The mesenteric lesion, profiled by a dashed line in (A), was examined under white light. (C) On the fluorescence image in the same view, PpIX-induced red fluorescence was detected in the mesenteric nodules. (D) The observed GFP-induced green fluorescence was consistent with the PpIX-induced fluorescence. Scale bar, 1 mm.

To investigate whether 5-ALA administration can specifically visualize peritoneal disseminations, a mouse model of human colon cancer was used. This model develops peritoneal disseminations in the abdominal cavity, which are microscopically visible within 2 weeks after the tumor implantation surgery. EGFP fluorescent positive nodules were considered to be metastatic, and 5-ALA-induced red fluorescence colocalized with these nodules (Fig. 1). Fluorescence spectra with a peak of ∼635 nm were also observed in these nodules using a spectral analytic system (data not shown).

### Laparoscopic diagnosis using 5-ALA in colorectal cancer patients

**Figure 2.**
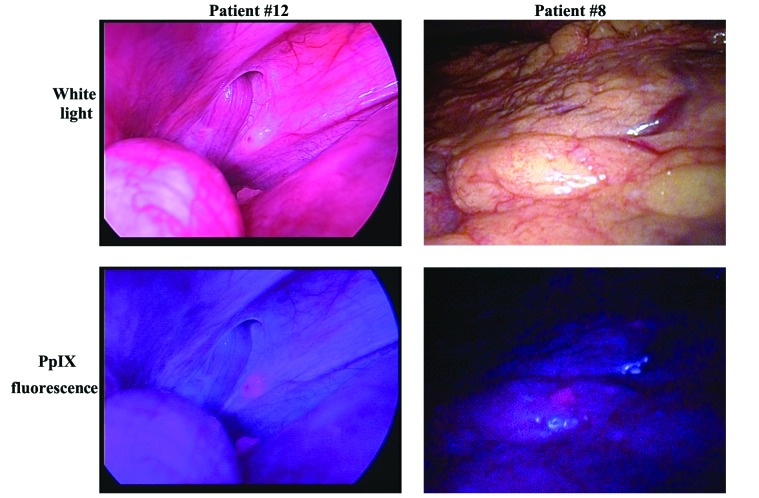
A laparoscopic image of the peritoneal metastases under white light (top) and PpIX-induced fluorescence (bottom). The red fluorescence observed was consistent with nodules suspected to be peritoneal metastases under white light observation (left). This case was observed with a D-LIGHT system. One small lesion, which was difficult to identify under white light, was easily detected with PpIX fluorescence observation (right).

The details of the patient characteristics are summarized in Table I. There were 9 men and 3 women (age range, 39–84 years). None of the enrolled patients experienced any side effects from the 5-ALA administration. All patients underwent laparoscopic observation first. Four patients underwent sigmoidectomy; 3 patients underwent right hemicolectomy; 1 patient underwent left hemicolectomy; 1 patient underwent transverse colectomy and 2 patients underwent explorative laparotomy with ileostomy. Eight patients underwent laparoscopic surgery, and 4 patients underwent laparotomy. Peritoneal dissemination was observed in 8 patients with conventional white light observation. All nodules suspected as peritoneal dissemination by white light observation were similarly detected in the fluorescence images (Fig. 2, left; this case was observed using a D-LIGHT system). Some fluorescent nodules were biopsied and pathologically confirmed as metastases (Table II). Liver metastases exposed on the liver surfacppe were observed in 6 patients using white light observation and fluorescence observation. In 1 patient, a small, flat lesion that was invisible under white light observation was only detectable by fluorescence imaging (Fig. 2, right). This nodule was biopsied and pathologically diagnosed as a peritoneal metastasis. Among the non-metastatic lesions, the fat tissue, liver, and bowel wall were observed as slightly redder than the abdominal wall.

### Quantitative analysis

**Figure 3.**
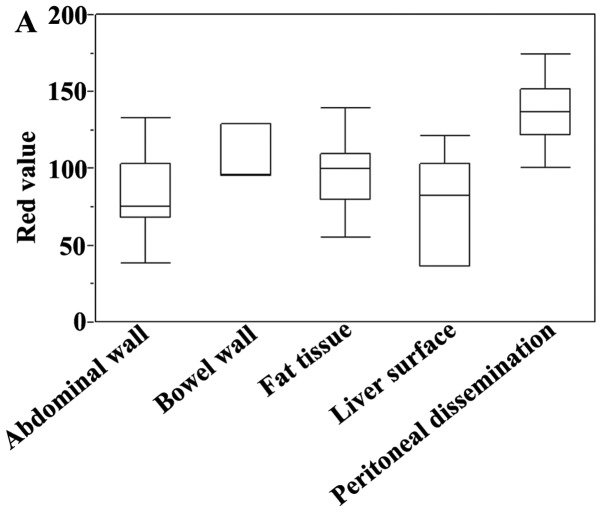

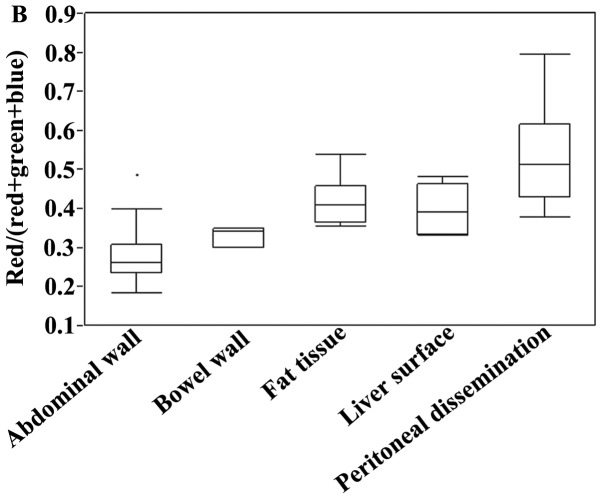
Box-and-whisker plots of a quantitative analysis of 5-ALA-induced fluorescence images. The red value of each 24-bit RGB color image was evaluated with a corresponding index for red fluorescence. The maximum red value of each peritoneal metastatic nodule and non-metastatic site of the abdominal wall, fat, and liver were compared (A). The red/(red + green + blue) ratio was also evaluated to correct for differences in the imaging conditions (B).

The 5-ALA-induced fluorescence images were analyzed. The maximum red value of each peritoneal metastatic nodule and representative non-metastatic lesions (bowel wall, fat tissue, and liver surface) were evaluated (Fig. 3A). The red value was higher in the metastatic nodules than in the non-metastatic lesions, but there was some variability. The red/(red + green + blue) ratio was significantly higher in the metastatic nodules than in the abdominal wall (P<0.001), bowel wall (P=0.0066), fat tissue (P=0.0057), and liver surface (P= 0.0014) (Fig. 3B). The ratio had less variability than the absolute red value.

## Discussion

Peritoneal dissemination is a form of colon cancer metastasis. Its prognosis is poor, and it is difficult to diagnose. Untreated peritoneal dissemination is associated with poor survival, and systemic chemotherapy alone does not appear to yield any clinically significant survival benefits (16,17). Of the patients diagnosed with colorectal cancer metastases, ≤25% have peritoneal dissemination without any other metastases (18,19). Diagnosing minimal peritoneal dissemination improves the prognosis because the patients undergo chemotherapy treatment earlier. However, a diagnosis of peritoneal dissemination is limited by the CT scanning, magnetic resonance imaging (MRI), and PET-CT detection limits, as well as the limitations of the surgeon’s gross inspection. Therefore, the development of a new, accurate diagnostic method is needed.

Various cancer-specific fluorescent probes have been developed. These probes have several advantages: they are minimally invasive; the machine parts are compact, and they allow for real-time diagnosis. However, clinically useful fluorescent probes are limited. 5-ALA has few side effects and is a safe drug that has previously been used to diagnose glioma and bladder cancer (7–10). In urology, the sensitivity of detecting dysplasia or early bladder cancer with fluorescence cystoscopy (96.9%) is significantly higher than that for white light cystoscopy (72.7%) (7). Conventional white light cystoscopy does not provide adequate information about the presence of ‘flat’ urothelial lesions, such as carcinoma *in situ*, but fluorescence cystoscopy reveals carcinoma lesions that do not look suspicious under white light cystoscopy (8). In neurosurgery, survival after surgery and radiotherapy for malignant glioma is linked to the completeness of tumor removal. Nonetheless, it is difficult to grossly differentiate malignant lesions from normal brain tissues, and serious complications may occur after extensive surgical removal. 5-ALA induced fluorescence can be used to visualize malignant glioma intraoperatively, and it allows for safer and more thorough tumor removal than conventional white light surgical treatments (9,10). By applying 5-ALA-PDD, the exact mapping of the malignant lesions and the visualization of less visible lesions have improved the therapeutic effect (20). Although 5-ALA-PDD is a useful method, no previous reports have used 5-ALA-PDD to diagnose peritoneal metastasis in colorectal cancer.

In this study, all nodules suspected to be peritoneal disseminations when they were observed by white light also emitted 5-ALA-induced red fluorescence. Of those red nodules, 8 were biopsied and diagnosed as metastatic. Moreover, 1 small nodule that was difficult to detect under white light was detected by 5-ALA-PDD. 5-ALA-PDD improved the diagnostic accuracy of the peritoneal dissemination of colorectal cancer by detecting small and/or flat nodules that are invisible under white light observation. Because 5-ALA-PDD allows for the diagnosis of peritoneal dissemination in real time during surgery, without requiring biopsy, it may be a useful diagnostic method for completing resecting cancer lesions and decreasing the occurrence of incomplete surgeries. 5-ALA-PDD is expected to be useful in diagnosing peritoneal metastasis in the early stages, thereby improving the treatment outcomes. Among the non-metastatic sites, the bowel wall, liver surface, and fat tissue were grossly observed as slightly reddish, but the suspected metastatic nodules in those sites were redder, making them distinguishable from the surrounding tissue. The reddish coloration of non-metastatic sites may be caused by the physiological accumulation of PpIX and/or its optical properties.

The evaluation of red fluorescence for 5-ALA-PDD has a subjective component. Therefore, we used an RGB image analysis to evaluate whether quantitative diagnosis is possible. We assigned the red value as a corresponding index for the 5-ALA-induced fluorescence intensity. The red value was significantly higher in the peritoneal metastases than in the non-metastatic lesions, but there was some variability because of the differences in the imaging conditions, particularly the brightness. The red + green + blue value corresponds to the brightness; thus, an evaluation of the red/(red + green + blue) ratio could be used to more clearly distinguish between peritoneal metastases and non-metastatic lesions than an evaluation of the red value alone.

This study has some limitations. It was difficult to inspect the entire peritoneal cavity with the rigid scope that we used in this study. To resolve this issue, in the near future, we will use a flexible scope that is capable of fluorescence observation. Another problem is the undesirable effect of the physiological accumulation of PpIX and autofluorescence of the surrounding tissues. In this study, we could detect nodules on the surface of fat tissue and liver, but the contrast was lower than that for other sites. Tissue autofluorescence sometimes interferes with the detection of PpIX fluorescence. If the autofluorescence of the tissue adjacent to a tumor nodule is strong, the red fluorescence of PpIX will not be detectable. New methods to reduce these effects are required to improve the diagnostic accuracy of 5-ALA-PDD.

In conclusion, we observed better diagnostic accuracy using 5-ALA-PDD compared to conventional laparoscopy in patients with colorectal cancer. 5-ALA-PDD is a promising candidate for diagnosing peritoneal dissemination in colorectal cancer.

## Figures and Tables

**Table I.** Enrolled patients.

Case	Gender	Age (years)	Tumor location	Histology	cT	cN	cM	Operation	
1	Male	82	S	tub2>por2	4a	1b	1a PER	Sigmoidectomy	Laparotomy
2	Male	39	S	tub1>tub2	4a	1b	1a PUL	Sigmoidectomy	Laparoscopic surgery
3	Female	47	Ra	tub1	4a	2a	1a PER	Ileostomy	Laparotomy
4	Male	43	S	Por2>tub1	4a	2b	1b HEP LYM	Sigmoidectomy	Laparoscopic surgery
5	Male	66	S	tub1	4a	0	0	Sigmoidectomy	Laparoscopic surgery
6	Male	62	D	tub1>tub2	4a	2a	0	Left hemicolectomy	Laparoscopic surgery
7	Male	84	S	tub1>tub2	4a	2a	1a HEP	Right hemicolectomy	Laparoscopic surgery
8	Male	69	S	tub1>tub2	4a	2b	1b HEP LYM OTH	Ileostomy	Laparoscopic surgery
9	Female	55	A	tub2>tub1	4a	1b	1b HEP PUL	Right hemicolectomy	Laparoscopic surgery
10	Male	72	T, S, RS	tub1>tub2	4a	2b	1a PER	Hartmann	Laparotomy
11	Male	74	T	tub2>tub1	4a	1b	1a HEP	Right hemicolectomy	Laparoscopic surgery
12	Female	45	T	tub2>por2	4a	1a	1a OTH	Transverse colectomy	Laparotomy

A, Ascending colon; T, Transverse colon; D, descending colon; S, sigmoid colon; RS, rectosigmoid; Ra, rectum above the peritoneal reflection. tub1, well differentiated adenocarcinoma; tub2, moderately differentiated adenocarcinoma; por2, poorly differentiated adenocarcinoma non-solid type.

**Table II.** Comparison of 5-ALA mediated fluorescence laparoscopic imaging and pathological examination.

	Examination by FL		Pathology	
Case	Peritoneal dissemination	Liver metastasis	Depth of invasion	Peritoneal dissemintion
1	+	+	SE	+
2	+	+	SI (bladder)	+
3	+	−		+
4	−	−	SE	−
5	−	−	SS	−
6	−	−	SS	−
7	+	+	SS	+
8	+	+		+
9	−	+	SE	−
10	+	−	SS	+
11	+	+	SE	+
12	+	−	SE	+

SS, tumor invades subserosa; SE, tumor perforates serosa; SI, tumor extending to adjacent organs; FL, fluorescence laparoscopy.

## References

[1] Sugarbaker PH (1995). Peritonectomy procedures. Ann Surg.

[2] Glehen O, Gilly FN, Boutitie F, Bereder JM, Quenet F, Sideris L, Mansvelt B, Lorimier G, Msika S, Elias D (2010). Toward curative treatment of peritoneal carcinomatosis from nonovarian origin by cytoreductive surgery combined with perioperative intraperitoneal chemotherapy: a multi-institutional study of 1,290 patients. French Surgical Association Cancer.

[3] Brücher BL, Piso P, Verwaal V, Esquivel J, Derraco M, Yonemura Y, Gonzalez-Moreno S, Pelz J, Königsrainer A, Ströhlein M, Levine EA, Morris D, Bartlett D, Glehen O, Garofalo A, Nissan A (2012). Peritoneal carcinomatosis: cytoreductive surgery and HIPEC - overview and basics. Cancer Invest.

[4] Koppe MJ, Boerman OC, Oyen WJG, Bleichrodt RP (2006). Peritoneal carcinomatosis of colorectal origin. Ann Surg.

[5] Bamba Y, Itabashi M, Kameoka S (2012). Clinical use of PET/CT in peritoneal carcinomatosis from colorectal cancer. Hepatogastroenterology.

[6] Ohgari Y, Nakayasu Y, Kitajima S, Sawamoto M, Mori H, Shimokawa O, Matsui H, Taketani S (2005). Mechanisms involved in delta-aminolevulinic acid (ALA)-induced photosensitivity of tumor cells: relation of ferrochelatase and uptake of ALA to the accumulation of protoporphyrin. Biochem Pharmacol.

[7] Kriegmair M, Baumgartner R, Knuchel R, Stepp H, Hofstadter F, Hofstetter A (1996). Detection of early bladder cancer by 5-aminolevulinic acid induced porphyrin fluorescence. J Urol.

[8] Jichlinski P, Forrer M, Mizeret J, Glanzmann T, Braichotte D, Wagnieres G, Zimmer G, Guillou L, Schmidlin F, Graber P, van den Bergh H, Leisinger HJ (1997). Clinical evaluation of a method for detecting superficial surgical transitional cell carcinoma of the bladder by light induced fluorescence of protoporphyrin IX following the topical application of 5-aminolevulinic acid: preliminary results. Lasers Surg Med.

[9] Stummer W, Stocker S, Wagner S, Stepp H, Fritsch C, Goetz C, Goetz AE, Kiefmann R, Reulen HJ (1998). Intraoperative detection of malignant gliomas by 5-aminolevulinic acid-induced porphyrin fluorescence. Neurosurgery.

[10] Friesen SA, Hjortland GO, Madsen SJ, Hirschberg H, Engebraten O, Nesland JM, Peng Q (2002). 5-Aminolevulinic acid-based photodynamic detection and therapy of brain tumors (review). Int J Oncol.

[11] Hatakeyama T, Murayama Y, Komatsu S, Shiozaki A, Kuriu Y, Ikoma H, Nakanishi M, Ichikawa D, Fujiwara H, Okamoto K, Ochiai T, Kokuba Y, Inoue K, Nakajima M, Otsuji E (2013). Efficacy of 5-aminolevulinic acid-mediated photodynamic therapy using light-emitting diodes in human colon cancer cells. Oncol Rep.

[12] Hino H, Murayama Y, Nakanishi M, Inoue K, Nakajima M, Otsuji E (2013). 5-Aminolevulinic acid-mediated photodynamic therapy using light-emitting diodes of different wavelengths in a mouse model of peritoneally disseminated gastric cancer. J Surg Res.

[13] Murayama Y, Harada Y, Imaizumi K, Dai P, Nakano K, Okamoto K, Otsuji E, Takamatsu T (2009). Precise detection of lymph node metastases in mouse rectal cancer by using 5-aminolevulinic acid. Int J Cancer.

[14] Murayama Y, Ichikawa D, Koizumi N, Komatsu S, Shiozaki A, Kuriu Y, Ikoma H, Kubota T, Nakanishi M, Harada Y, Fujiwara H, Okamoto K, Ochiai T, Kokuba Y, Takamatsu T, Otsuji E (2012). Staging fluorescence laparoscopy for gastric cancer by using 5-aminolevulinic acid. Anticancer Res.

[15] Koizumi N, Harada Y, Murayama Y, Harada K, Beika M, Yamaoka Y, Dai P, Komatsu S, Kubota T, Ichikawa D, Okamoto K, Yanagisawa A, Otsuji E, Takamatsu T (2013). Detection of meta-static lymph nodes using 5-aminolevulinic acid in patients with gastric cancer. Ann Surg Oncol.

[16] Klaver YLB, Lemmens VEPP, Creemers GJ, Rutten HJT, Nienhuijs SW, de Hingh IHJT (2011). Population-based survival of patients with peritoneal carcinomatosis from colorectal origin in the era of increasing use of palliative chemotherapy. Ann Oncol.

[17] Verwaal VJ (2003). Randomized trial of cytoreduction and hyperthermic intraperitoneal chemotherapy versus systemic chemotherapy and palliative surgery in patients with peritoneal carcinomatosis of colorectal cancer. J Clin Oncol.

[18] Maggiori L, Elias D (2010). Curative treatment of colorectal peritoneal carcinomatosis: current status and future trends. Eur J Surg Oncol.

[19] Cao C, Yan TD, Black D, Morris DL (2009). A systematic review and meta-analysis of cytoreductive surgery with perioperative intraperitoneal chemotherapy for peritoneal carcinomatosis of colorectal origin. Ann Surg Oncol.

[20] Zöpf T, Schneider AR, Weickert U, Riemann JF, Arnold JC (2005). Improved preoperative tumor staging by 5-aminolevulinic acid induced fluorescence laparoscopy. Gastrointest Endosc.

